# Impact of Polyphenol Supplementation on Energy Expenditure Measured by Indirect Calorimetry in Adolescents with Metabolic Dysfunction-Associated Steatotic Liver Disease: A Pilot Randomized Study

**DOI:** 10.3390/healthcare13243215

**Published:** 2025-12-08

**Authors:** Christine Haïkal, Marie-Catherine Turcotte, Véronique Bélanger, Sophia Morel, Anik Cloutier, Emile Levy, Valérie Marcil, Ramy El-Jalbout, Véronique Groleau

**Affiliations:** 1 Azrieli Research Center of the CHU Sainte-Justine, Montreal, QC H3T 1C5, Canada; christine.haikal@umontreal.ca (C.H.); marie-catherine.turcotte.med@ssss.gouv.qc.ca (M.-C.T.); veronique.belanger.hsj@ssss.gouv.qc.ca (V.B.); sophia.morel@umontreal.ca (S.M.); anik.cloutier2.hsj@ssss.gouv.qc.ca (A.C.); levy.emile@gmail.com (E.L.); ramy.el-jalbout.med@ssss.gouv.qc.ca (R.E.-J.); veronique.groleau.med@ssss.gouv.qc.ca (V.G.); 2 Department of Nutrition, Faculty of Medicine, Université de Montréal, Montreal, QC H3T 1J4, Canada; 3 Department of Gastroenterology, CHU Sainte-Justine, Montreal, QC H3T 1C5, Canada; 4 Department of Pediatrics, Faculty of Medicine, Université de Montréal, Montreal, QC H3T 1J4, Canada

**Keywords:** polyphenols, indirect calorimetry, metabolic dysfunction-associated steatotic liver disease, adolescent, metabolism

## Abstract

Background: Metabolic dysfunction-associated steatotic liver disease (MASLD) is increasingly prevalent among adolescents, especially those with obesity. It is the leading cause of liver-related morbidity and mortality and can progress to metabolic dysfunction-associated steatohepatitis, and eventually irreversible cirrhosis. There is currently no medical treatment recommended for MASLD in adolescents. Nutritional interventions, such as polyphenol supplementation, could be a non-pharmacological option to improve metabolic outcomes. Objectives: This pilot study aimed to preliminarily assess the impact of a 60-day polyphenol supplementation on measured resting energy expenditure (mREE) by indirect calorimetry (IC) in adolescents with MASLD. It also compared mREE by IC with predicted resting energy expenditure (pREE) using the WHO and Schofield formulae. Methods: This single-blind randomized controlled trial enrolled 23 adolescents with MASLD, of which 11 completed IC assessments before and after the 60-day polyphenol supplementation (intervention group, n = 5) or no supplementation (controls, n = 6). There was no placebo. Caloric intake was assessed to evaluate its impact on mREE and mREE was compared to pREE using the WHO and Schofield equations. Results: Participants in the intervention group had a statistically significant increase in mREE between the two visits (+89.6 kcal/day, *p* = 0.037), while no difference was found in the control group. When compared to the control group, the intervention group had a greater variation in mREE between visits (+100.4 kcal/day, *p* = 0.021). No significant changes were observed when adjusting mREE for body weight. Also, there were no significant changes in body weight in the two groups between the visits. Both the WHO and Schofield equations overestimated pREE with an average percentage of pREE of 88.8% and 91.0%, respectively. Conclusions: Although several methodological limitations prevent clear conclusions from being drawn at this stage, this study suggests that polyphenol supplementation could increase REE in adolescents with MASLD and that the WHO and Schofield equations tend to overestimate REE in obese patients.

## 1. Introduction

Metabolic dysfunction-associated steatotic liver disease (MASLD), defined by steatotic liver disease related to systemic metabolic dysregulation, has become more frequent in adolescents in the last decade [1,2,3]. It is usually associated with other metabolic disorders like obesity, insulin resistance, hypertension, dyslipidemia, and type 2 diabetes [4]. The prevalence of MASLD in patients with severe obesity may exceed 90% and up to 69% in patients with type 2 diabetes [1]. Obesity can be explained, among other factors, by genetic, metabolic, socioeconomic, behavioral, and environmental factors [5] and has numerous physical and psychological consequences for children and adolescents [6,7,8]. It is an important risk factor for developing cardiovascular diseases like type 2 diabetes, arterial hypertension, and MASLD. Different studies have demonstrated that infantile obesity is a key indicator of adult obesity, and that early intervention can reduce its risk in the long term [9,10]. Nutritional interventions in youths could help them maintain a healthy weight and reduce the risk of associated chronic diseases.

MASLD is the most frequent liver disease worldwide and is the leading cause of liver-related morbidity and mortality [3,11]. It affects both adults and children with an increasing prevalence; from 26% before 2005 to 38% in more recent studies [4]. The same tendency is observed in adolescents, with a prevalence of less than 3% in 1990 and more than 10% today [12]. Children with a family medical history of type 2 diabetes or liver steatosis are more at risk of developing MASLD [13,14]. Other contributing factors like intestinal dysbiosis and genetic variants have also been described [13]. If not treated or identified early, MASLD can progress to metabolic dysfunction-associated steatohepatitis (MASH), characterized by the presence of fibrosis, and eventually irreversible cirrhosis, and as a risk factor for hepatocellular carcinoma in adults [15]. Interestingly, a recent study highlighted a decreasing tendency in the mortality rates for children with MASLD, suggesting that early detection and interventions for diabetes and obesity may improve the outcomes of these patients [16].

There is currently no medical treatment recommended for MASLD [17]. The recommendations are weight loss through diet modification and physical activity [18]. A prospective study conducted from 2009 to 2013 demonstrated that patients with weight losses of more than 10% presented with the highest rates of non-alcoholic fatty liver disease activity score reduction, MASH resolution, and fibrosis regression [19]. Because few patients achieve this goal, there is a need for other therapeutic options before considering bariatric surgery or liver transplant. Current treatments used for MASLD are vitamin E and antidiabetic agents like pioglitazone. However, vitamin E cannot be recommended for the treatment of MASLD because of a lack of evidence on the long-term efficacy and safety [1]. As for pioglitazone, adverse effects limit its use [20]. Obethicolic acid, resmetirom, and aramchol are under phase 3 randomized controlled trials for the treatment of non-cirrhotic MASH [21] and resmetirom was recently approved by the FDA for adults with biopsy-confirmed non-cirrhotic MASH. Unfortunately, there is currently no data on the long-term efficacity and safety of these treatments, and even less so in children. In one of the few trials conducted in children, vitamin E was compared to metformin and placebo with a primary outcome of sustained reduction in ALT levels; neither vitamin E nor metformin was found to be superior to placebo [22].

Polyphenols are natural compounds synthesized by plants with chemical features related to phenolic substances that have antioxidant, anti-inflammatory, and anticancer properties [23,24,25,26,27], and exhibit the capacity to modify intestinal microbiota [28,29]. They are mainly found in fruits and vegetables, cereals, chocolate, green tea, and wine. Studies have demonstrated therapeutic effects of polyphenols in obesity management through the regulation of fat metabolism and adipogenesis [30,31]. Recent studies have demonstrated that a polyphenol-rich diet could contribute to MASLD prevention and treatment by increasing fatty acid oxidation and modulating insulin resistance, oxidative stress, and inflammation [18,31,32,33,34,35,36,37,38]. The impact of polyphenols on resting energy expenditure (REE) [39], measured by indirect calorimetry (IC), was also evaluated in clinical trials. While some studies showed promising results with an increase in REE or in fatty acid oxidation [40,41,42,43,44], others were inconclusive [45,46]. Hence, the authors of a systematic review of studies evaluating the effect of catechins on fat metabolism, basic metabolic rate (BMR), REE, and respiratory quotient (RQ) could not reach a definite conclusion because of conflicting results [47]. However, all these studies were conducted in adults and no data are yet available in adolescents, for whom such an intervention could prove beneficial in the long term.

REE can be estimated using a predictive formula. Multiple formulae exist to estimate REE using anthropometric data, age, sex, and fat free mass, and may be used to properly prescribe nutritional therapy [48]. The most-used predictive formulae are the World Health Organization (WHO) formula, the Harris–Benedict formula, the Schofield formula based on weight, the Schofield formula based on weight and height, and the Oxford formula [49]. The accuracy of these equations has been assessed in different pediatric populations in the past including in healthy children [50] and adolescents [51], as well as in obese and non-obese children and adolescents [48,49,52].

Finally, REE can be modulated by caloric intake. Excessive caloric consumption increases REE while fasting, while a restrictive diet tends to decrease it [53]. Also, REE after weight loss is lower than expected according to body composition, promoting weight regain [54]. Hence, for people with obesity, a supplement increasing REE could be an interesting tool for weight management.

Here, we present a pilot sub-study of a feasibility trial on the effects of polyphenol supplementation on hepatic steatosis, intima-media thickness, and non-invasive vascular elastography in obese adolescents [55]. The aim is to preliminarily assess, in adolescents with MASLD, the impact of polyphenol supplementation on energy expenditure measured by IC. We also aim to compare measured REE (mREE) by IC with predicted REE (pREE) using the WHO and Schofield formulae.

## 2. Methods

### 2.1. Study Design and Participants

This study took place within the frame of a prospective open-label randomized controlled feasibility trial without placebo at the CHU Sainte-Justine in Montreal, Canada, for which the protocol has been described [55]. The trial was single-blind, i.e., the research team (researchers, coordinators, research professionals, and gastroenterologist performing the IC) was not aware of the allocation of the participants’ group. Study participants were not blinded to the intervention group. Inclusion criteria were as follows: age between 12 and 18 years old, body mass index (BMI) percentile > 85th for age and sex, and biopsy-confirmed or clinical diagnosis of MASLD. Exclusion criteria were as follows: being pregnant; having a known chronic systemic disease; having a serious condition that would prevent compliance and safe participation to the study; taking antibiotics, vitamins—excluding vitamin D—or natural supplements; weight loss of 5–10% of the usual weight in the last 6 months before recruitment or weight change of 5% in the last 3 months; alcohol consumption of >2 drinks/day or >1 day/week; and known allergies to ingredients in the polyphenol supplement and/or known peanut allergy.

Participants were recruited from a generated list of eligible patients from the CHU Sainte-Justine liver biopsy and hepatology clinic registry. The GraphPadstatistical system (https://www.graphpad.com/quickcalcs/, last accessed on 15 July 2021) was used for simple randomization to allocate participants to treatment and control groups. The polyphenol supplement consisted of a concentrate of 67 polyphenols from elderberries, honeyberries, wild blueberries, aronia berries, and blackcurrants in liquid form (commercial polyphenol, specific composition of medicinal ingredients available in Supplementary Table S1). Participants randomly allocated to the treatment group were asked to take a single dose of 5 mL (17,230 mg of the polyphenol concentrate) every morning before breakfast for 60 days. Compliance was verified through weekly phone calls and filling out a daily logbook. Participants allocated to the control group received no treatment or placebo. IC was performed at 2 visits: before the initiation of treatment and after 60 days of treatment. Written consent from all participants and parents were obtained, and the study was approved by the institutional research scientific and ethics boards (#2020-2278, 12 March 2021). Clinical data were collected at both visits and included age, sex, weight, and height. The blind was lifted after all patients completed the study to allow for data analysis.

### 2.2. Indirect Calorimetry

REE (kcal/day) was measured at both visits by a research professional trained by a pediatric gastroenterologist specialized in IC using an open-circuit canopy IC with a computerized metabolic cart (Vmax Encore, Vyaire medical, Yorba Linda, CA, USA). The same cart was used for all IC assessments. Assessments were performed in the morning and participants were required to fast (12 h). During the assessment, participants were resting and awake in a semi-reclined supine position for 60 min. Participants were allowed to watch a movie during the assessment to stay awake. Data collected during IC assessments were interpreted by the pediatric gastroenterologist. The first ten minutes and any periods of significant movement that correlate with changes in REE were excluded from the edited assessment. Remaining data points were averaged and the REE was calculated from the modified Weir equation. Achievement of steady state was defined as a coefficient of variation (CV) of less than 5%. REE is presented in kcal/day and in kcal/kg/day.

### 2.3. Assessment of Caloric Intake

Participants’ caloric intake was analyzed to assess the stability of caloric consumption during the intervention and to examine its impact on REE. Nutritional intake was collected using food records and 24 h dietary recalls. For the first 17 participants, daily food records were collected throughout the intervention. Given the high burden of this method, a modification to the protocol was made for participants 18 to 23 and dietary data were collected using 24 h dietary recalls. To assess caloric intake before each visit, an average of the two most complete days from the food journals in the week preceding the visit was calculated, or one 24 h recall for the day previous to the visit was used. Nutritional data were analyzed using Nutrific^®^ Software (version 1.1) developed by the Department of Food Science and Nutrition of Laval University and based on the 2015 Canadian Nutrient File.

### 2.4. Analysis

For continuous variables, means and standard deviations for normally distributed data were calculated, and medians and ranges were used for skewed data. For categorical variables, frequency distributions were used. Participants’ clinical characteristics and calorimetry data were analyzed with the Wilcoxon or Mann–Whitney test to compare distributions. For analyses in within groups, paired *t*-tests were used while independent *t*-tests were employed to compare two groups. Pearson correlation was used to determine the relationship between variations in caloric intake and mREE between visits. Predicted REE (pREE) with the Schofield and WHO equations was compared to measured REE (mREE) via IC by calculating the percentage of mREE/pREE. These equations are defined in Supplementary Table S2. An overestimation of REE with the equations was defined as a percentage of mREE/pREE of 90% or less. Associations between caloric intake and EE were assessed with the Pearson correlation coefficient. Results were considered statistically significant at *p* < 0.05. SPSS (IBM, Armonk, NY, USA, version 28.0.1.0) was used for statistical analysis.

## 3. Results

### 3.1. Study Flowchart and Characteristic of Cohort

**Figure 1:**
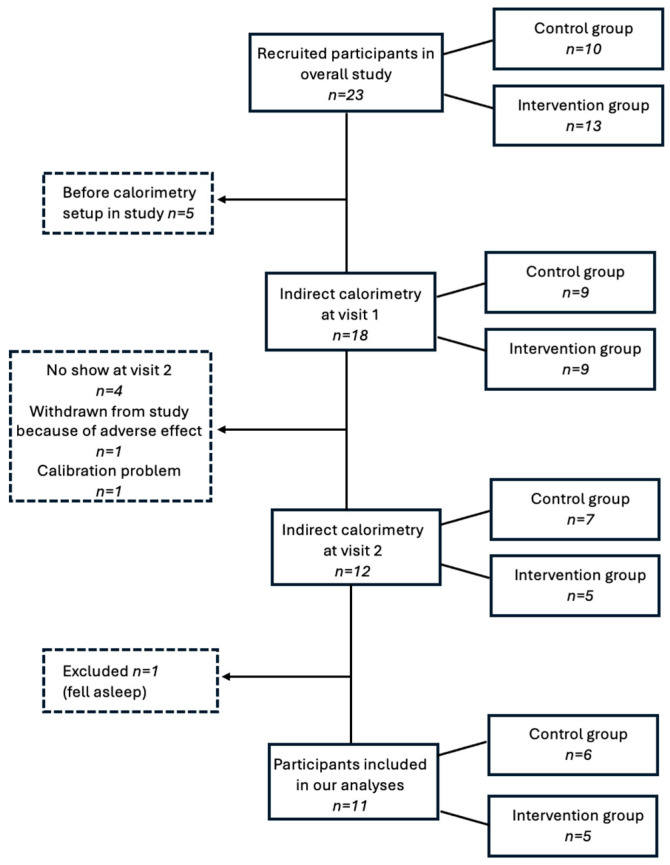
Study flowchart.

The study flowchart is presented in Figure 1.

Overall, 23 participants (78.2% male) were recruited in the study (characteristics presented in Supplementary Table S3), of which 10 were allocated to the intervention group and 13 to the control group. Mean age at recruitment was 14.8 years (range 12.1 to 17.9 years) and mean BMI was 35.6 kg/m^2^ (range 25.4 to 48.0 kg/m^2^). Adverse events are detailed in Supplementary Table S4. A total of 18 participants completed the IC assessment at visit 1, as the calorimetry experimental set-up was not initiated before the sixth participant for logistical reasons. Of those, four did not come to the second visit, one was withdrawn from the study because of an adverse event (i.e., loss of appetite), although it was considered unrelated to the supplement, and one was excluded because of a calibration problem during the IC assessment. Therefore, IC assessment was performed in 12 participants, of which 1 was excluded for analysis because he fell asleep during the first assessment, which considerably lowered his mREE and thus introduced an important bias. Data were analyzed in a total of 11 participants, of which 6 had been assigned to the control group and 5 to the intervention group. Clinical characteristics of the final subgroup at both visits are presented in Table 1, in addition to caloric intake data and IC measurements.

Mean time between visits was 62.5 ± 5.5 and 72.8 ± 12.1 days for the control and intervention groups, respectively (*p* = 0.94). For all participants in the intervention group, the duration of the supplementation was 60 days. An assessment of compliance revealed an average number of days with supplement intake of 54.8 days (minimum: 48 days; maximum: 60 days). There was no difference between the control and intervention groups in terms of age, weight, height, BMI, and caloric intake. Of note, between the two visits, in each group, there was no statistically significant difference in mean weight nor BMI, while participants in the intervention group were slightly taller at their second visit (Table 1).

### 3.2. Relation Between mREE and Energy Intake

Mean calorie intake for the intervention and control groups is presented in Table 1. The results show that, in each group, there was no statistically significant difference in caloric intake between the two visits. Also, we found no relationship between the variation in caloric intake and mREE between visits (r of Pearson= −0.215; *p* = 0.526).

### 3.3. Comparison of Participants’ mREE Between Visits in the Control and Intervention Groups

The mean total duration of the IC assessment ranged between 57.8 and 60.4 min and was between 27.6 and 33.2 min after editing (Table 2).

Steady state (CV < 5%) was achieved for all participants at both visits. When analyzing the difference between mREE at visits 1 and 2, there were no statistically significant difference in controls. In the intervention group, there was an increase of 89.6 kcal/day (*p* = 0.037) after the supplementation period (Table 2). However, when mREE was adjusted for participants’ weight, the increase was not statistically significant.

### 3.4. Variation in mREE Between Visits in the Control and Intervention Groups

**Figure 2:**
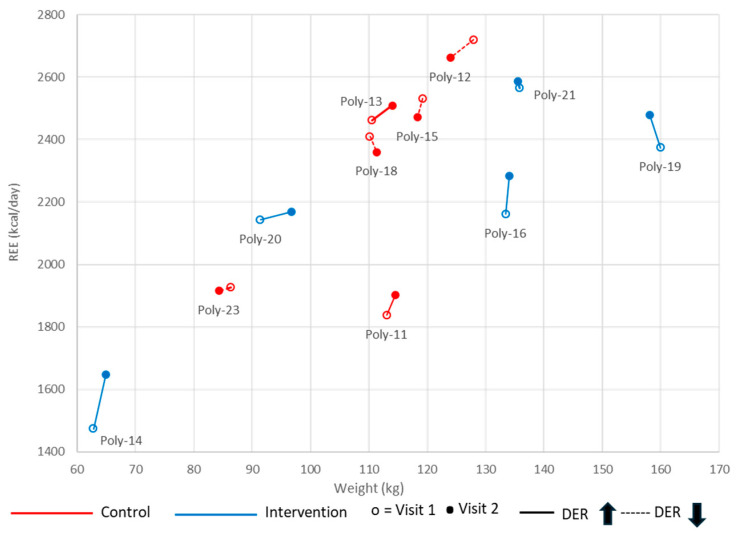
Measured resting energy expenditure (REE) in relation to weight between visits 1 and 2. Participant’s REE and weight were measured at visits 1 (empty dot) and 2 (full dot). An increase is identified by a solid line, and a decrease by a dashed line. Data are shown for each group: in red for the control group and in blue for the intervention group.

Figure 2 illustrates the change in REE relative to weight between the two visits in the control (red) and intervention (blue) groups. The coordinates (x = weight, y = REE) are shown for each participant at visits 1 and 2 to visualize mREE in relation to weight over time and highlight the variability in individual patterns between visits. Participants labeled Poly-12, Poly-15, Poly-18, and Poly-23 show a decrease in REE (dotted line), while those labeled Poly-11, Poly-13, Poly-14, Poly-16, Poly-19, Poly-20, and Poly-21 show an increase in REE (solid line) from visit 1 to visit 2.

Comparing the mean variation in mREE among visits (V2-V1) between the intervention and control groups showed a higher variation in mREE in participants who received the polyphenol supplementation, with a mean difference in mREE variation of 100.43 ± 71.00 kcal/day (*p* = 0.021) (Table 3). However, although the variation in weight-adjusted REE remained higher in the supplementation group than in controls (0.60 ± 0.42 kcal/kg/day), the difference was not statistically significant. Also, when adjusting REE and weight-adjusted REE for caloric intake, the variation was not statistically significant.

### 3.5. Comparison of mREE with pREE

We compared mREE with pREE calculated using the WHO and Schofield equations to evaluate the adequacy of these equations in obese adolescents with MASLD (Table 4). On average, the WHO and Schofield equations overestimated mREE (%mREE/pREE = 88.8% and 91.0%, respectively). Accordingly, for most measures taken, mREE was <100% of pREE (21 out of 22 assessments for the WHO equation, 95.4%; and 20 out of 22 assessments for the Schofield equation, 90.9%). No statistical significance was found between the two equations for either %mREE/pREE or for the proportion of assessments in the corresponding intervals. For the two equations, there was no difference between the control and polyphenol groups at V1 and at V2.

## 4. Discussion

The findings of this pilot study support the positive impact of a 60-day polyphenol supplementation on energy expenditure in adolescents with obesity and MASLD, this without an impact on weight or BMI. This study also highlights the tendency for the WHO and Schofield equations to overestimate mREE in this population. To our knowledge, this is the first study investigating the impact of polyphenol supplementation on REE in adolescents. While several methodological limitations prevent clear conclusions from being drawn at this stage, the results of this study can be used for effect estimation to determine sample size in future clinical trials.

When comparing results with the literature, it is important to consider that all published studies were performed in adults supplemented with various polyphenols formulations, doses, and study durations, which limits comparability. Moreover, the impact of an increase of 89 kcal per day in REE may not be clinically significant. Nonetheless, our results suggesting a stimulating effect of polyphenols on REE (mean of +89.6 kcal/day) align with a meta-analysis that found a significant increase in 24 h EE in participants receiving a supplementation of a mixture of catechin–caffeine and of only caffeine compared to placebo (mean of 428.0 kJ [102.3 kcal] and 429.1 [102.36 kcal], respectively) [40]. Similarly, a randomized controlled trial demonstrated that green tea extract (containing EGCG and caffeine) increased 24 h EE by 3.5% (corresponding to 329 kJ [78.6 kcal]) compared to placebo in ten healthy men [42]. Although both studies used IC, their methodology differed from ours, as 24 h EE rather than REE was measured. Another study using IC with a canopy found that a thermogenic supplement containing catechin and caffeine increased REE for at least 4 h post-ingestion in moderate caffeine consumers (ranging from 123.4 to 147.3 kcal/day) [41]. Furthermore, a double-blind, randomized crossover trial in 18 overweight participants aged 20–50 years showed that short-term supplementation with EGCG and resveratrol increased REE compared to the placebo group (mean of 1873 ± 60 vs. 1798 ± 61 kcal/day, i.e., a difference of 75 kcal/day) [56]. In contrast, a placebo-controlled randomized trial involving 60 patients who took EGCG and caffeine capsules for 12 weeks found no difference in REE measured by IC between groups or over time in the polyphenol group [45]. Also, resveratrol supplementation in 11 healthy obese men reduced REE measured in a respiratory chamber, contradicting our findings [57]. Additionally, in a randomized, double-blind, placebo-controlled crossover trial in 12 non-obese men, catechin- and caffeine-rich oolong tea had no impact on 24 h EE [46]. These differences underline the importance of standardizing research protocols for each of the sub-populations studied. At this stage, there is a real need to study pediatric and adolescent populations in whom such nutraceutical approaches could be beneficial in the long term.

REE, adjusted for weight, refers to the energy expenditure at rest expressed per kilogram of body weight (kcal/kg/day). REE naturally increases with body weight and adjusting it can help compare REE between individuals of different sizes. In our setting, no statistically significant difference was observed between the intervention and control groups when mREE was adjusted for body weight. As such, adjusting REE for lean body mass and fat-free mass removes body composition-associated biases and should be considered in obese patients [58]. The fact that these measurements were not available in our study could explain the results. As a matter of fact, in obese populations, weight alone is an imprecise indicator for metabolically active tissue because individuals with similar body weights can differ substantially in their proportion of FFM versus fat mass. Without data from direct body composition measurements (e.g., dual-energy X-ray absorptiometry, bioelectrical impedance analysis), it is not possible to determine whether the observed differences in REE reflect true metabolic changes or simply underlying variations in FFM between participants or across visits. This limitation reduces the precision of our interpretation and may partly confound associations between polyphenol supplementation and REE. Future studies should include FFM assessment to enable more accurate normalization of REE and to better isolate the metabolic effects of the intervention.

No statistically significant difference was observed in participants’ caloric intake between the beginning and end of the intervention. Accordingly, no association between energy intake and REE was found. In the literature, studies have demonstrated a reduction in REE following a hypocaloric diet [59,60], supporting an impact of caloric intake on REE. In a study assessing the effect of daily caloric intake on REE in 100 participants aged 18 to 25 years, a weak correlation between daily caloric intake and REE [61] was observed. In our study setting, given that the caloric intake remained relatively stable during the study, it would not appear to be an important confounding factor. However, data on habitual polyphenol consumption were not available, which could confound the results and affect the internal validity of the intervention.

Finally, when comparing the WHO and Schofield pREE with mREE by IC, an overestimation of REE was found. Even if the accuracy of these equations has been assessed in obese children and adolescents in the past [59], recent studies have demonstrated that previously developed predictive equations provide inaccurate estimates of REE [49,62,63,64]. Our results align with these findings and call for caution in their use with this population.

Our study has several strengths. The use of a randomized, single-blind design minimizes bias, strengthening the validity of the results, and increasing the likelihood that the observed effects are attributable to the intervention. Additionally, despite the pilot nature of the study, it is, to our knowledge, the first to assess the impact of a combination of polyphenols on REE in adolescents with MASLD. IC, the gold standard for REE measurement, ensures accurate and reliable measurements.

However, important limitations must be acknowledged. The small sample size limits statistical power and may affect the ability to detect significant differences between groups. The lack of placebo in the trial is a major limitation and could introduce an important bias, in particular with regard to the monitoring of participants’ compliance. Also, the 60-day intervention period may be insufficient to observe long-term effects and/or to evaluate sustained changes in EE. Given that the polyphenol supplement combined different polyphenols, it is not possible to identify which specific compounds would be responsible for the observed metabolic effects. The use of two different methods to assess nutrition intake limits the ability to compare between participants. Moreover, important confounding variables impacting REE were not collected in the framework of our study. These include fat-free mass, genetic factors, pubertal stage, baseline metabolic status, physical activity, sleep patterns, menstrual cycle phase in females, baseline metabolic status, smoking status, and dietary polyphenol intake. Not adjusting for these cofactors, in addition to the methodological design not allowing for the establishment of a causal relationship, prevents reliable conclusions from being drawn regarding the impact of polyphenols on REE. This will need to be considered in future clinical trials.

## 5. Conclusions

In conclusion, our study suggests that polyphenol supplementation could increase REE in adolescents with MASLD, although several methodological shortcomings prevent clear conclusions from being drawn at this stage. Future research is needed to better understand the role of polyphenols in energy metabolism. Overall, this pilot study contributes to advancing the understanding of the impact of polyphenols on REE and opens avenues to use them as a tool for weight management.

## Figures and Tables

**Table 1:** Comparison of clinical characteristics and caloric intake of participants in control and intervention groups at both visits.

Caracteristics	Visit 1		Visit 2	
	Control	Intervention	Control	Intervention
Male/female, n	4/2	4/1	—	—
Age (y), mean ± SD	15.3 ± 1.6	14.2 ± 1.6	15.5 ± 1.6 ***	14.4 ± 1.6 **
Weight (kg), mean ± SD	111.2 ± 13.9	116.7 ± 38.9	111.1 ± 13.8	117.9 ± 36.9
Height (cm), mean ± SD	173.4 ± 9.8	175.1 ± 10.6	173.7 ± 9.7	175.5 ±10.5 *
BMI (kg/m^2^), mean ± SD	37.1 ± 5.2	37.3 ± 9.5	37.0 ± 5.4	37.5 ± 8.7
Caloric intake (kcal/day), mean ± SD	1837 ± 250	2189 ± 377	1990 ± 425	2752 ± 990

Measured resting energy expenditure (mREE) was assessed using an open-circuit canopy indirect calorimetry with a computerized metabolic cart. Caloric intake was calculated based on the average of the two most complete days from the food journals the week before the visit or one 24 h recall of the day before the visit. Mean differences between visits 1 and 2 (V2-V1) were compared with paired *t*-tests. A *p* value < 0.05 was considered statistically significant. mREE: measured resting energy expenditure; SD: standard deviation. * *p* = 0.031; ** *p* < 0.01; *** *p* < 0.001 vs. visit 1.

**Table 2:** Comparison of indirect calorimetry assessment data and measured resting energy expenditure of participants in control and intervention groups at both visits.

Characteristics	Visit 1		Visit 2	
	Control	Intervention	Control	Intervention
Indirect calorimetry assessment				
Total duration (min.), mean ± SD	57.8 ± 5.2	57.8 ± 5.6	59.2 ± 4.8	60.4 ± 1.9
Edited duration (min.), mean ± SD	33.2 ± 8.1	27.6 ± 3.6	29.0 ± 10.8	32.6 ± 6.0
CV (%), mean ± SD	3.6 ± 1.2	4.4 ± 1.1	3.9 ± 0.5	3.8 ± 1.0
Measured resting energy expenditure				
mREE (kcal/day), mean ± SD	2314 ± 352	2143 ± 412	2304 ± 321	2233 ± 366 *
Weight-adjusted mREE (kcal/kg/day), mean ± SD	21.0 ± 2.3	19.4 ± 4.0	20.8 ± 2.2	19.9 ± 4.0

Measured resting energy expenditure (mREE) was assessed using an open-circuit canopy indirect calorimetry with computerized metabolic cart. The edited assessment excluded the first ten minutes and any periods of significant movement. Mean differences between visits 1 and 2 (V2-V1) were compared with paired *t*-tests. A *p* value < 0.05 was considered statistically significant. mREE: measured resting energy expenditure; CV: coefficient of variation; SD: standard deviation. * *p* = 0.037 vs. visit 1.

**Table 3:** Mean variation in mREE between the two visits in the control and intervention groups.

Indirect Calorimetry Assessment		Groups				
	n	Control	n	Intervention	Mean Differences ΔmREE (kcal/d) ± SD	*p* Value
Δ mREE (kcal/d), mean (min-max)	6	−10.83 (−60–64)	5	89.60 (23–174)	100.43 ± 71.0	0.021
Δ Weight-adjusted mREE (kcal/kg/d), mean (min-max)	6	−0.05 (−0.68–0.41)	5	0.55 (−1.33–1.88)	0.60 ± 0.42	0.238

The resting energy expenditure (REE) was measured by indirect calorimetry and the variation between the two visits was assessed. The mean differences in measured resting energy expenditure between the two visits (V2-V1) between the two groups (control and intervention) were assessed by independent *t*-test. A *p* value < 0.05 was considered statistically significant. mREE: measured resting energy expenditure; SD: standard deviation.

**Table 4:** Percentages of measured REE relative to predicted REE calculated using the WHO and Schofield equations at visits 1 and 2.

	WHO		Schofield	
	V1	V2	V1	V2
**% mREE/pREE** (mean ± SD) **n = 11**	88.1 ± 9.5	89.4 ± 9.2	90.3 ± 9.9	91.7 ± 10.1
**Mean (%)**	88.8		91.0	
**Corresponding interval**, n (%)				
61–70	1 (9)	0 (0)	1 (9)	0 (0)
71–80	1 (9)	2 (18)	1 (9)	2 (18)
81–90	3 (27)	5 (46)	2 (18)	3 (27)
91–100	6 (55)	3 (27)	6 (55)	5 (46)
101–110	0 (0)	1 (9)	1 (9)	1 (9)

The percentage of resting energy expenditure (REE) measured by indirect calorimetry relative to the REE predicted using the WHO and Schofield equations was calculated. mREE: measured resting energy expenditure; pREE: predicted resting energy expenditure; WHO: World Health Organization; SD: standard deviation; V1: visit 1; V2: visit 2.

## Data Availability

Available upon reasonable request.

## Supplementary Materials

The following supporting information can be downloaded at https://www.mdpi.com/article/10.3390/healthcare13243215/s1. Table S1: Specific composition of medicinal ingredients of the polyphenol supplement (per 5 mL); Table S2: Estimation equations; Table S3: Characteristics of all participants included in the study; Table S4: Summary of adverse events.
