# Supplementary material for: Impact of Polyphenol Supplementation on Energy Expenditure Measured by Indirect Calorimetry in Adolescents with Metabolic Dysfunction-Associated Steatotic Liver Disease: A Pilot Randomized Study
Source: Healthcare (Basel). 2025 Dec 8;13(24):3215. doi: 10.3390/healthcare13243215 (PMC12733041; doi:10.3390/healthcare13243215)
Supplement: Supplementary file 1 [file healthcare-13-03215-s001.zip › healthcare-3950575-supplementary.pdf]

Supplementary Table S1. Specific composition of medicinal ingredients of the polyphenol supplement (per 5 ml)

Cold concentrate of:	Quantity
Canadian Elderberry (*Sambucus canadensis, fruit*)	5,417 mg
Haskap Berry (*Lonicera caerulea, fruit*)	4,958 mg
Wild Blueberry (*Vaccinium angustifolium, fruit*)	4,604 mg
Aronia Berry (*Aronia melanocarpa, fruit*)	2,063 mg
Black Currant (*Ribes nigrum, fruit*)	188 mg

N.B. As this is a natural health product, the product composition may vary and cannot be confirmed.

Supplementary Table S2. Estimation equations

**SCHOFIELD**	**Boy**<br>0-3 years old: (0.167 x weight (kg)) + (15.174 x height (cm)) – 617.6<br>3-10 years old: (16.59 x weight (kg)) + (1.303 x height (cm)) + 414.9<br>10-18 years old: (16.25 x weight (kg)) + (1.372 x height (cm)) + 515.5<br>>18 years old: (15.057 x weight (kg)) + (1.0004 x height (cm)) + 705.8<br>**Girl**<br>0-3 years old: (16.252 x weight (kg)) + (10.232 x height (cm)) – 413.5<br>3-10 years old: (16.969 x weight (kg)) + (1.618 x height (cm)) + 371.2<br>10-18 years old: (8.363 x weight (kg)) + (4.65 x height (cm)) + 200<br>>18 years old: (13.623 x weight (kg)) + (23.8 x height (cm)) + 92.2
**WHO**	**Boy**<br>0-3 years old: (60.9 x weight (kg)) - 54<br>3-10 years old: (22.7 x weight (kg)) + 495<br>10-18 years old: (17.5 x weight (kg)) + 651<br>**Girl**<br>0-3 years old: (61 x weight (kg)) - 51<br>3-10 years old: (22.5 x weight (kg)) + 499<br>10-18 years old: (12.2 x weight (kg)) + 746

WHO: World Health Organization

Supplemental Table S3. Characteristics of all participants included in the study

Characteristics	n	
Sex, n (%)		
Female	5	(22)
Male	18	(78)
Age (y), Mean (min – max)	23	14. 8 (12.1 – 17.9)
Weight (kg), mean (min – max)	23	108.1 (62.8 – 160.0)
Height (cm), mean (min –max)	23	173.7 (157.2 – 188.5)
BMI (kg/m^2), mean (min – max)	23	35.6 (25.4 – 48.0)

Supplementary Table S4. Summary of adverse events

	**Intervention group (n=13)**
**Participants with AE reported, n (%)**	7 (53.8%)
**Total AE reported[1], n**	34
**Severity[2]**	
**Severe, n (%)**	0 (0%)
**Moderate, n (%)**	11 (32.4%)
**Mild, n (%)**	23 (67.6%)
**Relation[2]**	
**Related, n (%)**	0 (0%)
**Suspected, n (%)**	7 (20.6%)
**Not related, n (%)**	27 (79.4%)
**AE expected[2]**	
**Yes, n (%)**	6 (17.6%)
**No, n (%)**	28 (82.4%)

[1]**Adverse effect reported included**: Nausea, vomiting, headache, stomach pain, cold, cough, runny nose, throat irritation, breathing difficulty, tiredness, muscle pain, gastritis, decrease in appetite, change in smell, dry throat, blood in urine, redness and itching, crying, dizziness, pimples.

[2]Percentage of AE on total AE reported.
